# Melanin Pigment in Plants: Current Knowledge and Future Perspectives

**DOI:** 10.3389/fpls.2020.00770

**Published:** 2020-06-23

**Authors:** Anastasiia Y. Glagoleva, Olesya Y. Shoeva, Elena K. Khlestkina

**Affiliations:** ^1^Cereal Functional Genetics Group, Institute of Cytology and Genetics, Siberian Branch of the Russian Academy of Sciences, Novosibirsk, Russia; ^2^N.I. Vavilov All-Russian Research Institute of Plant Genetic Resources (VIR), Saint Petersburg, Russia

**Keywords:** seed, diagnostic physicochemical tests, melanoplast, polyphenol oxidase, enzymatic browning reaction

## Abstract

The word “melanin” refers to a group of high molecular weight, black, and brown pigments formed through the oxidation and polymerization of phenolic compounds. This pigment is present in all kingdoms of living organisms, but it remains the most enigmatic pigment in plants. The poor solubility of melanin in particular solvents and its complex polymeric nature significantly constrain its study. Plant melanin synthesis is mostly associated with the enzymatic browning reaction that occurs in wounded plant tissues. This reaction occurs when, due to the disruption of cellular compartmentation, the chloroplast-located polyphenol oxidases (PPOs) release from the chloroplast and interact with their vacuolar substrates to produce *o*-quinones, which in turn polymerize to melanin. Furthermore, the presence of melanin in intact seed tissues has been demonstrated by diagnostic physicochemical tests. Unlike the well-studied enzymatic browning reaction, little is known about how melanin is formed in seeds. Recent data have shown that it is a tightly controlled genetic process that involves many genes, among which the genes encoding PPOs might be key. The present article aims to provide an overview of the current knowledge on melanin in plants and to discuss future perspectives on its study in light of recent findings.

## Introduction

Brown and black seed color is a widespread trait in plants. The color can be caused by melanin, which is a high molecular weight pigment formed by the oxidation and polymerization of phenols (Britton, 1985; Solano, 2014). It is present in all kingdoms of living organisms but remains hitherto the most enigmatic pigment in plants. The lack of scientific attention to this plant pigment is due to the absence of obvious functions that might be ascribed to it (Thomas, 1955). For a long time, this plant pigment was not considered to be melanin since, according to the definition of the term “melanin,” which was formulated based on explorations of melanin in animals, it must be a nitrogen-containing pigment; melanin in plants does not contain nitrogen (Thomas, 1955; Prota, 1992). Comparative studies of the black pigments extracted from microorganisms, plants, and animals revealed their common physicochemical properties except for the presence of nitrogen (Nicolaus et al., 1964). The terminology was reconsidered, and the requirement for nitrogen was excluded from the definition of the term “melanin” (Britton, 1985; Solano, 2014). Currently, three types of melanin are recognized: eumelanins, pheomelanins, and allomelanins. Eumelanins are predominant forms found in animals and microorganisms, and occur in some fungi; pheomelanins are specific of higher animals, mammals, or birds. Both of them are derivatives of tyrosine, but pheomelanins consist of sulfur-containing monomeric units, mostly benzothiazine and benzothiazol, instead of indole units in eumelanins. Plant and fungal melanin, devoid of nitrogen is generically named as allomelanin (other melanins). It is the most heterogeneous group; its precursors are varied. Fungal melanin can be formed from gamma-glutaminyl-3,4-dihydroxybenzene, catechol, and 1,8-dihydroxynaphthalene, while catechol, caffeic, chlorogenic, protocatechuic, and gallic acids are considered to be the possible precursors in plants (Lyakh, 1981; Bell and Wheeler, 1986; Solano, 2014). Due to the unique features of melanin, such as its stable free radical state, ultraviolet-visible (UV-Vis) light absorption, and complexation and ion-exchange capacities, these pigments have attracted growing interest as materials for a broad range of biomedical and technological applications (d’Ischia et al., 2015; Di Mauro et al., 2017; Vahidzadeh et al., 2018). Since plant melanin is present in most cases in low-cost agricultural waste products (e.g., grape pomace and sunflower seed husks), it has attracted special attention. The potential of melanin from sunflower husks as a sorbent with high enterosorption efficiency and as an antiaging agent in elastomer compositions has been demonstrated (Gracheva and Zheltobryukhov, 2019; Kablov et al., 2019).

In comparison with those in animals and microorganisms, the biochemical and molecular-genetic aspects of melanin formation in plants have been less studied. One of the reasons, in addition to the complex polymeric nature of the pigment, is that plant melanin accumulates in hard seed envelopes where other compounds with similar colors, such as proanthocyanidins, can be present. It seems clear that the starting point of any biochemical and molecular-genetic study of melanogenesis in plants is to confirm the melanic nature of the pigment. To evaluate the current state of research on plant melanogenesis and outline future research directions, in this review, we gathered data on the functions, localization, and molecular-genetic control of melanin formation in seeds with an emphasis on studies in which the melanic nature of the pigment was proven by physicochemical methods.

## Physicochemical Methods To Identify and Study Plant Melanins

The standard protocol of melanin detection includes their alkaline extraction and subsequent precipitation in acid conditions (Sava et al., 2001). Extracted this way pigment material represents a dark glossy powder, which is insoluble in water and in the most organic solvents, partially soluble in concentrated sulfuric and nitric acids, and fully soluble in sodium hydroxide. When exposed to strong oxidizing agents, such as hydrogen peroxide, potassium permanganate, or bromine water, the pigment loses its color, while exposure to ferric chloride results in the precipitation of a flocculent material that gradually redissolves when the concentration of ferric chloride is raised. The results of the reactions indicate the presence of quinoid and phenolic groups in melanins (Thomas, 1955; Fox and Kuchnow, 1965; Lyakh, 1981; Downie et al., 2003; Shoeva et al., 2020).

In addition to chemical tests, spectroscopic techniques have been applied to confirm the melanic nature of pigments. UV-Vis spectroscopy is the most broadly used to identify and quantify melanins. Melanins of different origin are characterized by high absorbance in visible and ultraviolet spectrum with the maximum at 196–300 nm (Lyakh, 1981; Pralea et al., 2019). To identify the major functional groups in the melanin macromolecules, Fourier transform infrared (FT-IR) spectroscopy has been used. The typical FT-IR spectra of melanin include characteristic bands for phenolic fragments, quinone, aliphatic hydrocarbon groups, and an aromatic carbon backbone (Mbonyiryivuze et al., 2015; Pralea et al., 2019). Nuclear magnetic resonance (NMR) analysis can be used to confirm the presence in melanins aromatic hydrogens and carbons, methyl, or methylene groups attached to nitrogen and/or oxygen atoms, NH-group linked to indole, alkyl fragments (Pralea et al., 2019). Melanins are paramagnetic biopolymers due to the presence of stable free radicals, which can be detected by electron paramagnetic resonance (EPR) spectroscopy (Butterfield, 1982). A characteristic EPR signal of melanins is attributed to semiquinone radicals (Enochs et al., 1993).

**Figure 1:**
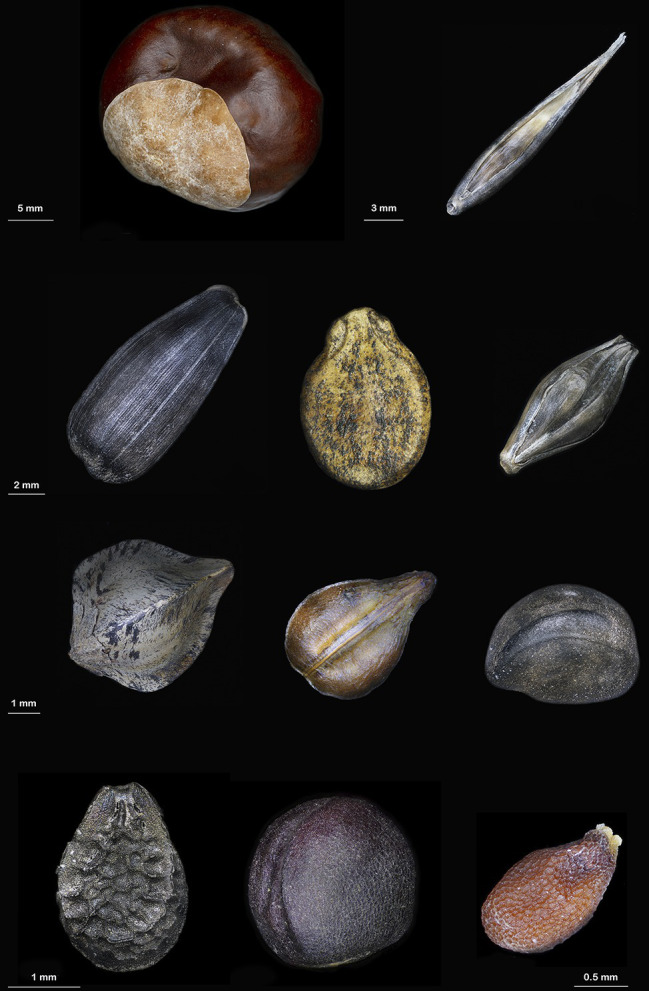
Some plant species accumulate melanins in seeds; the presence of melanins was confirmed by physicochemical methods. First row (from left to right): chestnut (*Castanea mollissima*) and oat (*Avena sativa*), second row: sunflower (*Helianthus annuus*), watermelon (*Citrullus lanatus*), and barley (*Hordeum vulgare*), third row: buckwheat (*Fagopyrum esculentum*), grape (*Vítis vinífera*), and ipomoea (*Ipomoea purpurea*), fourth row: sesame (*Sesamum indicum*), rape (*Brassica napus*), and black mustard (*Brassica nigra*).

Through the use of chemical tests in combination with some of the described spectroscopic techniques, the melanic nature of the black pigments in seeds has been proved for the following species: watermelon (Nicolaus et al., 1964), sunflower (Nicolaus et al., 1964; Gracheva and Zheltobryukhov, 2016), buckwheat (Zhuravel, 2010), grape (Zherebin and Litvina, 1991), tomato (Downie et al., 2003), fragrant olive (Wang et al., 2006), night jasmine (Kannan and Ganjewala, 2009), sesame (Panzella et al., 2012), ipomoea (Park, 2012), black mustard and rape (Yu, 2013), chestnut (Yao et al., 2012), garlic (Wang and Rhim, 2019), oat (Varga et al., 2016), and barley (Shoeva et al., 2020; Figure 1). Promising results in determining the structure of plant melanins were recently obtained by matrix-assisted laser desorption/ionization-time of flight mass spectrometry (MALDI-TOF MS), that was applied to resolve the structure of oat melanin, which turned out to be a homopolymer built up from *p*-coumaric acid and consists mainly of low molecular weight oligomers of 3–9 monomer units (Varga et al., 2016).

Although melanins were confirmed in the seeds of a few plant species, the fact that these species belong to distinct taxonomical groups implies a wider distribution of the pigments than has currently been demonstrated.

## The Functions of Melanin Pigments in Plants

It is believed that black pigmentation arose as a result of the adaptation of living organisms to unfavorable environmental conditions. The functional importance of this type of pigment has been reviewed in detail for animals, insects, and microorganisms (Solano, 2014; Cordero and Casadevall, 2017). The role of the pigment in plants is still vague, but the gathered information demonstrates that the black color might grant some advances to them as well.

As in animals, melanin-based coloration in plants is important for camouflage. For instance, most wild cereals have black hull pigmentation. Falling to the ground when mature, the seeds covered by black hulls are considered to be invisible to birds on a background of dark soil (Zhu et al., 2011).

Due to the ability of black surfaces to absorb more solar energy than light surfaces and convert it to heat, theoretically, black-grained seeds can mature earlier than yellow seeds. A comparative study of barley landraces with black and white seeds demonstrated that the former tend to mature earlier than the latter (Ceccarelli et al., 1987).

Melanins provide additional mechanical strength to seed shells, protecting them from damage. Moreover, melanin provides resistance to insects and pests due to its toxicity (Jana and Mukherjee, 2014). In sunflower, seeds with black seed coats are less damaged by mole larvae than white seeds (Pandey and Dhakal, 2001).

As melanins are strong antioxidants (Panzella et al., 2012; Lopusiewicz, 2018), they can confer more vigor to seeds that accumulate them and can protect seeds under stress. There are some examples to support this hypothesis. In watermelon, the brown seeds were more vigorous than the light-colored seeds; they had higher seed weight, germination and emergence percentages, and seedling fresh and dry weight than light-colored seeds (Mavi, 2010). In *Brassica* species, yellow seeds with transparent seed coatings have thinner hulls and less fiber than varieties with dark, thicker, and more lignified seeds (Marles and Gruber, 2004). The local Syrian barley landraces with black seeds are grown in the most arid regions of the country, unlike the white-grained landraces that are adapted to milder growing conditions (Ceccarelli et al., 1987). A comparison of these samples showed that samples with black grains are more cold-and drought-tolerant than samples with white grains (Ceccarelli et al., 1987; Weltzien, 1988). Attempts to demonstrate the protective functions of melanin in barley grain under salinity, drought, and cadmium toxicity using a precise genetic model of near-isogenic lines (NILs) differing by grain color have been conducted. The data obtained demonstrated that melanin does not confer any advantages to barley seedlings under the stress conditions tested (Glagoleva et al., 2019). More convincing results on the protective functions of melanins were obtained while testing resistance to pathogen infection. Varieties of barley and oat with a dark spike color were less affected by *Fusarium* infection than varieties without dark husk pigments (Zhou et al., 1991; Loskutov et al., 2016). The barley recombinant inbred lines (RILs) with black grains demonstrated lower *Fusarium* head blight incidence and lower accumulation of the mycotoxin deoxynivalenol than RILs with yellow grains (Choo et al., 2015).

Compounds accumulating in seed envelopes are known to affect the dormancy and germination rate of seeds (Debeaujon et al., 2000; Gu et al., 2011). This is true in the case of flavonoid pigments, but some controversial results have been obtained in the case of melanin. For example, two tomato mutants with dark testa caused by melanin displayed a poor germination rate and percentage on both water and gibberellin compared with those of wild-type seeds in which melanin pigments were not detected (Downie et al., 2003). However, a comparative study of the germination rate of barley seeds of NILs with different grain colors did not reveal any differences between yellow and black grains (Glagoleva et al., 2019).

Based on the summarized data, one can conclude that melanins are not essential for plants. Therefore, it is likely difficult to reveal their functional role. However, the widespread distribution of this pigment implies its functional importance, which is yet to be identified in plants.

## Melanin Synthesis in Plants and Its Molecular-Genetic Control

**Figure 2:**
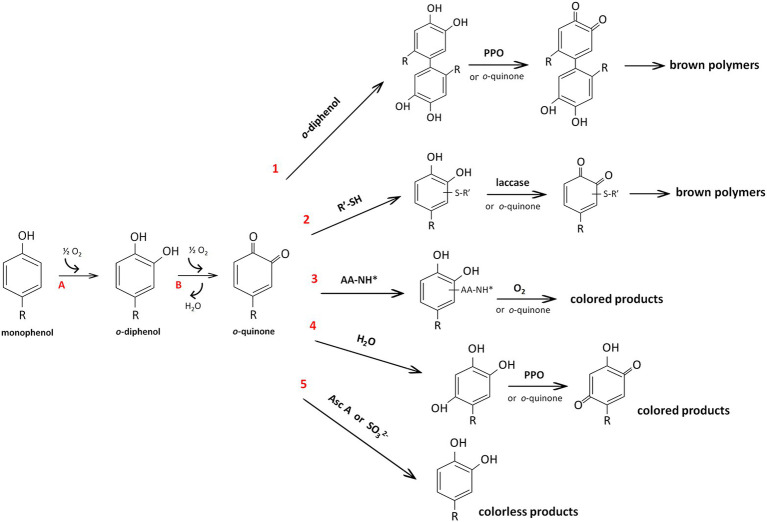
Reactions catalyzed by polyphenol oxidase (PPO) (A, and B) and reactions of *o*-quinone (1–6) according to Nicolas et al. (1994). Due to monophenolase (or cresolase) and diphenolase (or catecholase) activity, PPOs hydroxylate monophenols to *o*-diphenols (A) and subsequently oxidize *o*-diphenols to *o*-quinones (B), respectively. The resulting *o*-quinones can react with another molecule of phenol with the formation of dimers of the original phenol (reaction 1). These dimers with an *o*-diphenolic structure can be oxidized either enzymatically or by another *o*-quinone to a brown polymer. By nucleophilic addition, *o*-quinones can interact with thiol groups (reaction 2) or amino groups of amino acids or peptides (reaction 3), resulting in compounds with an *o*-diphenolic structure that can be further oxidized (by laccase or oxygen) or react with an excess of *o*-quinones to form colored products. Water can be added to *o*-quinones, leading to triphenols that can be oxidized by PPO or by *o*-quinones with the formation of *p*-quinones (reaction 4). Finally, the reactions with ascorbic acid or sulfites lead to the regeneration of the original phenol (reaction 5). All reactions are nonenzymatic except for those with laccase and PPO. AA-NH^*^, amino acids or peptides; Asc A, ascorbic acid; R’-SH, small thiol compounds (e.g., cysteine or glutathione).

Melanin synthesis in plants is associated with the enzymatic browning reactions that occur in damaged tissues by polyphenol oxidases (PPOs), which belong to a family of Cu-containing oxidoreductases that are able to act on phenols in the presence of oxygen (Nicolas et al., 1994). The loss of the integrity of cellular compartments due to senescence, wounding, interactions with pests and pathogens, or handling during postharvest processing and storage results in the release of PPOs from plastids where they are located into the cytoplasm. The PPOs come into contact with vacuolar phenolic substrates and form highly reactive *o*-quinones. The *o*-quinones subsequently either undergo nonenzymatic polymerization or interact with other compounds, such thiols, amino acids, and peptides, and form colored products; they can also slowly interact with water, resulting in the formation of triphenols or can be reduced to the original phenols (Figure 2). Since PPOs cause undesirable browning in plant products, the physicochemical properties of these enzymes have been studied in many economically important species, including *in vitro* studies of substrate specificity of the purified enzymes (Jukanti, 2017; Taranto et al., 2017). Nevertheless, PPOs remain one of the most intensively studied enzymes, since they are expected to have other functions besides the enzymatic browning reaction; of these possible functions, the functions related to their localization in chloroplasts are the most intriguing puzzle (Sullivan, 2014; Boeckx et al., 2015, 2017).

The participation of PPOs in melanin formation in intact seed tissues is in question. Until recently, melanin pigments were considered to accumulate extracellularly in the form of a phytomelanin layer. This would exclude the participation of plastid-located PPOs in melanin formation and implies some other phenol-oxidizing enzymes with extracellular localization as candidates for melanin synthesis, such as cell wall-associated laccases (Wang et al., 2015). However, recent observations of melanin accumulation in chloroplast-derived melanoplasts identified in black grains of barley (Shoeva et al., 2020), forces us to reconsider the association of melanin synthesis with the phytomelanin layer. The phytomelanin layer has been described as a black, hard, resistant material that fills intercellular spaces between the hypodermis and sclerenchyma in the pericarp of some sunflower family species (Pandey and Dhakal, 2001). The chemical structure of the material constituting the phytomelanin layer has not been defined. Some authors suggest that it is nonmelanic and consider it to be a derivative of a polyvinyl aromatic alcohol (Pandey and Dhakal, 2001; Jana and Mukherjee, 2014). However, the simultaneous presence of the phytomelanin layer and melanin in the seeds of some species, such as in the husks of sunflower plants (Thomas, 1955; Rogers and Kreitner, 1983; Gracheva and Zheltobryukhov, 2016), makes it difficult to distinguish these two terms. As melanin formation occurs intracellularly within plastids (Shoeva et al., 2020), and the extracellular phytomelanin layer is formed as a result of the catabolism of hypodermal cells (Pandey and Dhakal, 2001), it seems likely that melanin synthesis and phytomelanin layer formation are different cellular processes that should be distinguished.

Melanoplasts were discovered only in barley seeds, and additional studies on melanin-accumulating seeds are required to confirm melanin synthesis localization in this type of plastid. However, this finding, in addition to the data on the presence of the phenolic substrates of PPOs in chloroplasts (Zaprometov and Nikolaeva, 2003; Boeckx et al., 2017), suggests that PPOs are the main enzyme participating in plant melanogenesis in intact seed tissues. This hypothesis is supported by the molecular genetics data, which showed an association of the black color of seeds with PPO genes. For example, two complementary genes determining black pigmentation in rice hulls have been identified: *Ph1*, which encodes PPO, and *Bh4*, which encodes a tyrosine transporter (Fukuda et al., 2012). However, the melanic nature of the black pigment in rice seeds was not confirmed chemically; it could only be suggested based on the observed association. The gene encoding PPO has been recently identified as a candidate gene responsible for melanin pigmentation in watermelon seeds (Li et al., 2020).

In some other plant species, data on the mode of genetic inheritance are currently available. It was shown that the presence of the phytomelanin layer in sunflower achenes is a dominant trait that is controlled monogenically by the *Pml* gene (Johnson and Beard, 1977). Studies on the inheritance of the pigmentation pattern in three layers of sunflower pericarp also strongly support that the presence of the phytomelanin layer (the outer pericarp layer) is controlled by a single dominant gene (Mosjidis, 1982).

In barley, black spike color caused by melanin is under monogenic control by the *Blp* locus (Costa et al., 2001). Three dominant alleles, *Blp1.b*, *Blp1.mb*, and *Blp1.g*, conferring extreme black, medium black, and light black or gray colors, respectively, have been reported. The segregation ratio of 3:1 was reported for crosses between barleys with different seed pigmentation intensities (Woodward, 1941). To date, the *Blp* locus has been narrowed down to 21 genes, and a gene encoding purple acid phosphatase has been suggested as a candidate (Long et al., 2019).

Data on melanin metabolism in relationship to other metabolic processes taking place in plant seeds were obtained. It was shown that dark-colored barley seeds have higher contents of phenolic compounds and lignin than uncolored seeds. Therefore, it was suggested that melanin biosynthesis genes may be connected to phenylpropanoid-derived biosynthesis pathways such as those for flavonoids and lignins (Choo et al., 2005; Shoeva et al., 2016). A comparative transcriptome analysis performed using barley NILs with black and uncolored seeds demonstrated the influence of the dominant *Blp* allele on the expression of more than a thousand genes, among which phenylpropanoid and fatty acid biosynthesis genes were over-represented (Glagoleva et al., 2017). In *Ipomoea tricolor*, it has been shown that accumulation of melanins in the seed coat are under control of the same *ItIVS* gene, which encodes a transcription factor with the bHLH domain that regulates anthocyanin biosynthesis (Park, 2012). In tomato, an epistatic analysis of the *bks* mutant, which accumulates dark melanin pigments in the testa, in respect to *anthocyaninless* mutants that are impaired in anthocyanin synthesis demonstrated that *bks* is truly epistatic to the *anthocyaninless* mutants. The data imply that the black-seed phenotype is caused by a lesion in a gene required for a step before the flavonoid biosynthesis branch (Downie et al., 2003). As a support for this finding, flavonoid biosynthesis pathway genes were demonstrated to be uninvolved in the formation of melanin in barley (Shoeva et al., 2016). The examples demonstrate that comparative molecular genetics studies represent an effective means of understanding melanin synthesis in the context of the total metabolic processes occurring in plant tissues.

## Conclusions and Perspectives

In the past decade, the study of melanin synthesis in plants has advanced significantly. One of the achievements in this field has been the acceptance of the fact that melanins are broadly distributed in the plant kingdom. Although their presence in seed envelopes is still not associated with any obvious function, their wide distribution suggests the existence of some functions, among which protection against pathogens is the most probable. The discovery of the association of melanin synthesis with intracellular plastids can be recognized as another achievement in plant melanin research. Localization of melanin synthesis in plastids of grain envelopes has been demonstrated in only one species; additional studies on other plant species are required to confirm this finding. Moreover, the functional importance of the localization of PPOs in chloroplasts has long been an unsolved puzzle. Given the evidence, it seems likely that the presence of PPOs in chloroplasts is not an accident and may be directly connected to melanogenesis. At a minimum, such a connection should be explored.

## Author Contributions

AG wrote original draft of the manuscript, OS and EK developed its conceptualization. All authors reviewed and edited the manuscript.

## Conflict of Interest

The authors declare that the research was conducted in the absence of any commercial or financial relationships that could be construed as a potential conflict of interest.
